# What makes a theme park experience less enjoyable? Evidence from online customer reviews of Disneyland China

**DOI:** 10.3389/fpsyg.2023.1120483

**Published:** 2023-06-29

**Authors:** Shizhen Bai, Hao He, Chunjia Han, Mu Yang, Xinrui Bi, Weijia Fan

**Affiliations:** ^1^School of Management, Harbin University of Commerce, Harbin, China; ^2^Department of Management, Birkbeck, University of London, London, United Kingdom

**Keywords:** tourists’ dissatisfaction, online reviews, Shanghai Disney resort, topic modelling, sentiment (SEN) analysis

## Abstract

The aim of this study was to explore the causes of tourists’ lesser enjoyment of theme parks through an unsupervised machine learning approach—structural topic modelling. Specifically, the research extracted a comprehensive list of discussion topics about the travel experience of tourists through the analysis of 112,000 customer reviews released by visitors to the Shanghai Disney Resort from 16 June 2016 to 4 March 2022. Then, we used sentiment analysis to distinguish positive and negative topics and to explore the relationship between tourists who buy different ticket types and sentiments in negative topics. The results show that problems such as “parking,” “service attitude,” “recommendation feeling,” “experience comparison,” and “entrance” may be the main reasons for an unpleasant experience. In addition, we also found that when tourists travel in groups (e.g., via family tickets), customers feel unhappy because of parking and fast track problems. Moreover, when tourists travel alone or with small groups, staff service attitudes, experience comparisons, and entrance processes are the sources of greater concern. Our findings can help theme park managers to better understand the expectations of tourists and take effective measures to tackle issues causing customer dissatisfaction, and they can also contribute to theme park studies in tourism management.

## 1. Introduction

In the last decade, with the growth of people’s living standards, the tourism industry has been developing rapidly (Xue et al., 2017). Theme parks in particular attract over 500 million visitors globally each year (Association/AECOM, 2019, 2020), which has made pivotal contributions to the development of the tourism industry. With the advent of Web media, visitors are able to share their travel experiences through online platforms such as TripAdvisor (Heo and Lee, 2009; Luo et al., 2020; Albayrak et al., 2021; Yang et al., 2021; Bai et al., 2023). The utilization and analysis of the large amount of online review data has been found to have great importance for improving business performance as the analysis results provide significant insights into customers’ expectations and their experiences (Kim et al., 2015; Gao et al., 2018).

The extant research has shown that negative online reviews are valuable in terms of identifying issues and improving services such as hotels and restaurants in the tourism industry (Papathanassis and Knolle, 2011). Indeed, by paying more attention to negative reviews, it may be easier for managers to comprehend why customers find less enjoyment in specific aspects and thus resolve customer complaints promptly. Existing studies regarding negative online reviews cover businesses such as hotels (Wang and Chaudhry, 2018; Nan et al., 2019), museums (Su and Teng, 2018), restaurants (Li et al., 2020), and airlines (Augusto et al., 2019), but to the best of our knowledge, no study has focused on theme parks. Within the theme park context, a few studies have investigated the competitive advantages of different theme parks by analysing their online customer reviews (Luo et al., 2020; Albayrak et al., 2021). These studies demonstrate which aspects the customers of different theme parks care about the most and which aspects should be considered as competitive advantages. However, there is little research that explores tourists’ dissatisfaction and the potential reasons behind this in theme parks. Therefore, this study aims to understand the aspects that result in negative experiences when visiting theme parks and how these negative experiences differ among different customer types (i.e., depending on the different ticket type purchases). Choosing Disneyland in China as an examining case, this study applies an unsupervised machine learning approach, structural topic modelling (STM), and sentiment analysis to process the review text data.

## 2. Methodology

To analyse the review content, structural topic modelling, which is an unsupervised learning approach (Roberts et al., 2019), was applied to online customer reviews. This approach has been applied in a number of text mining studies in tourism management (e.g., Luo et al., 2020; Yang et al., 2021), and it was selected for this study due to its good performance on extracting topics (Roberts et al., 2019).

A web scraper using Python was programmed to collect all customer reviews between June 16, 2016 and March 4, 2022 on Shanghai Disney Resort from the Meituan platform.^1^ The Meituan platform is an online reservation platform. Users can use it to book meals or buy tickets, after which they can post comments and ratings. Our empirical analysis included all 106,072 customer reviews in the dataset, which is an effective representative sample for the domestic theme park due to the large volume of reviews. To investigate whether different customer types may influence the visiting experience, the study defined five customer types, namely, adult, college student, children, family, and others, based on the purchased tickets.

The dataset was processed by the following steps: (a) data cleaning that removed non-Chinese text such as numbers and punctuation, (b) preprocessing Chinese characters using the *jieba* package^2^ in Python, and (c) tokenisation. Next, we selected the number of the topics, K, which is a critical parameter of the STM. More specifically, we ran a set of potential *K*s, from 2 to 30, and chose *K* = 20 after optimizing the following metrics: the semantic coherence of the topics, held-out likelihood, and residuals. Based on the top words generated from the STM, two social science researchers were recruited to discuss and assign the topic labels and categories. From the most frequented words and a review of a significant volume of customer comments for each topic, conclusive labels and categories were assigned by the research team, as shown in Table 1.

**Table 1:** Topic summary.

Topic label	Topic proportions (%)	Top words
Topic category: service		
Entrance	5.43	身份证(ID card), 不好玩(not fun), 太棒了(fantastic), 两三个(two or three),服务周到(considerate service), 人不多(not many people), 带进去(bring in)
Service attitude	5.34	服务态度(service attitude), 一个半(one and a half), 一家人(family), 舞台剧(the living theater), 女孩子(girl), 摩托车(motorcycle), 大门口(doorway)
Staff	4.56	工作人员(staff), 通行证(pass), 性价比(cost-effective), 不耐烦(impatient), 九点半(9:30), 态度恶劣(bad attitude), 打招呼(say hello)
Fast pass	4.26	快速通道(fast pass), 工作日(working day), 一整天(all day), 女朋友(girlfriend), 几分钟(some minutes), 等待时间(waiting time), 花车游行(parade)
Parking	3.76	迪斯尼(Disney), 停车场(park), 全世界(whole world), 消费者(customer), 打电话(call up), 检票口(ticket gate), 好不容易(after all the trouble)
Topic category: theme		
Environmental conditions in Disney	10.16	迪士尼(Disney), 环境优美(beautiful environment), 所有人(all), 动画片(cartoon), 名不虚传(well-deserved reputation), 一日游(one-day trip), 越来越(more and more)
Theme games	5.83	飞越地平线(fly over the horizon), 雷鸣山漂流(Thunder Mountain Rafting), 七个小矮人矿山车(seven dwarfs mine cart), 加勒比海盗沉落宝藏之战(Pirates of the Caribbean Battle for the Sunken Treasure),地平线(horizon), 旋转木马(carousel), 小飞侠天空奇遇(Peter Pan Sky Adventures)
Leap over the horizon experience	4.10	飞跃地平线(leap over the horizon), 小矮人(dwarf), 第一个(first), 视觉效果(visual effect), 加勒比(Caribbean), 爱丽丝(Alice), 第一排(first row)
Extreme light wheel experience	3.46	极速光轮(extreme light wheel), 大学生(college students), 太爽了(so cool), 极力推荐(highly recommended), 很久队(long-time team), 好几遍(several times), 坐上去(sit up)
Pirates of the Caribbean experience	2.88	加勒比海盗(Pirates of the Caribbean), 小飞侠(Peter Pan), 十分钟(10 min), 多一点(more), 一部分(a part), 开开心心(happy), 世界级(world class)
Theme park introduction	2.63	度假区(resort), 身临其境(immersive), 小伙伴(friend), 人猿泰山(Tarzan),二维码(QR code), 前一天(the day before), 看不到(out of sight)
Children’s theme	2.47	小孩子(children), 小熊维尼(Winnie the Pooh), 白雪剬主(Snow White), 地铁站(subway station), 大多数(most), 节省时间(save time), 人会少(less people)
Roller coaster experience	1.64	过山车(roller coaster), 不虚此行(well worth the trip), 八点半(8:30), 玻璃杯(glass),总动员(general mobilization), 一如既往(as always), 四十分钟(40 min)
Topic category: general discussion		
Enjoyable feeling	11.17	挺好玩(pretty fun), 哈哈哈(ha ha ha), 很漂亮(very beautiful),游乐园(amusement park), 挺不错(pretty good), 一般般(so so), 啊啊啊(ah ah ah)
Peak season visit	8.49	人太多(too many people), 半小时(half-hour), 服务员(waiter), 排不上(cannot get), 玩不上(cannot play), 排好长(long queue), 每一项(each item)
Recommending feeling	8.24	强烈推荐(strongly recommended), 海盗船(pirate ship), 没什么(it is nothing), 没玩到(did not play), 长时间(nothing), 第二天(second day), 大部分(most)
Kids experience	4.32	小朋友(children), 第二次(second time), 一分钟(1 min), 下雨天(rainy day), 卡通人物(cartoon characters), 方便快捷(convenient and fast), 好开心(so happy)
Experience comparison	4.06	第一次(first), 人山人海(huge crowds of people), 游乐场(amusement park), 欢乐谷(happy valley), 一个多(more than one), 有意思(interesting), 五分钟(5 min)
Others	3.99	很开心(very happy), 好孩子(good children), 玩过来(play over), 小盆友(little friends), 舍不得(reluctant), 人满为患(overcrowded), 好朋友(good friends)
Holiday visit	3.21	节假日(holiday), 童话世界(fairy-tale world), 千万别(do not), 爆米花(popcorn), 强烈建议(strongly recommended), 第三次(third time), 寒暑假(winter and summer vacation)

**Figure 1:**
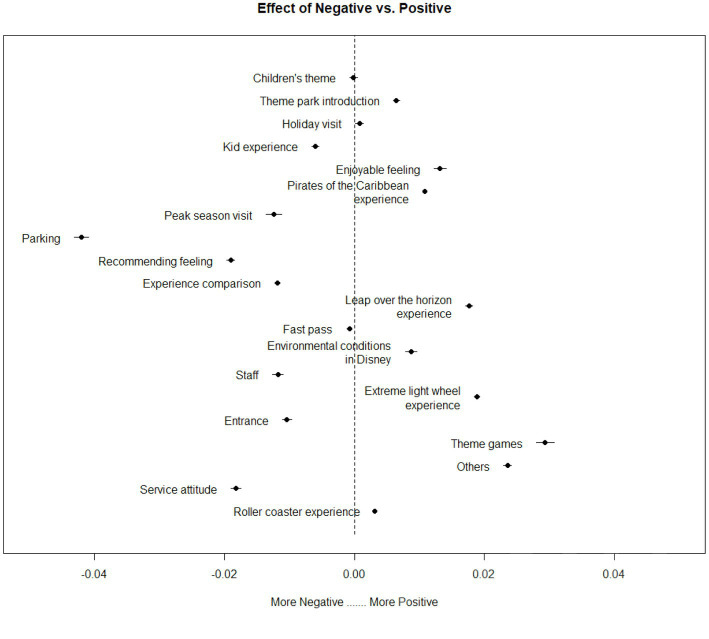
Negative vs. positive topics.

To obtain the negative topics, we firstly applied the sentiment analysis using the STM package (Roberts et al., 2019) in R, which calculated the sentiment score of each word in a review and then took the average score as the final sentiment score for each review. Next, the sentiment score of each topic was calculated by taking the overall sentiment of all the comments falling into the topic. In particular, a topic was identified as *negative* if the proportion of negative comments was larger than the proportion of positive comments. Conversely, if the proportion of positive comments was larger than the proportion of negative comments, then the topic was defined as a positive topic. The separation of topics based on the sentiment analysis is depicted in Figure 1.

## 3. Results and discussion

Based on the above analysis, 10 negative topics were identified, namely, Parking, Service Attitude, Recommending Feeling, Staff, Peak Season Visit, Experience Comparison, Entrance, Kids Experience, Fast Pass, and Children’s Theme. We compared these 10 negative topics with the results from a study by Albayrak et al. (2021), who conducted a comparative study on two theme parks, Ocean Park and Disneyland, in Hong Kong. They found that the disadvantages of Ocean Park were the “Staff,” “Fast pass,” and “F&B (Food and Beverage Services) and prices,” while the shortcomings of Disneyland in Hong Kong were “Child friendly,” “Waiting time,” “F&B and prices,” “Staff,” and “Accessibility.” Our findings also show that these aspects are likely to cause less enjoyment among theme park visitors. Specifically, the topics of “Staff” and “Fast pass” appeared in all three theme parks. In addition, the topics “Child friendly” and “Waiting time” identified in Hong Kong Disneyland are consistent with “Kids experience” and “Peak season visit” in Shanghai Disneyland. Moreover, our research reveals that the topics of “Parking,” “Service attitude,” “Experience comparison,” and “Entrance” could also be the reasons behind tourists’ dissatisfaction. This finding suggests that besides the child friendliness and waiting times, theme park managers may need to improve their parking and entrance facilities and services to match customer expectations.

Looking at the top three negative topics that have the highest proportions, the most negative topic is Parking, and the most frequent terms associated with this topic are *Disney*, *park*, *whole world*, *customer*, *call up*, *ticket gate*, and *after all the trouble*, showing that this topic primarily corresponds to parking services at Disneyland. Presented below is a visitor’s comment related to parking:

[Topic—Parking] I drove there. The parking fee was 100. Note that you only have to go in once and come out again, even if you have a receipt. What a hole! I picked up a friend and paid again, and the tickets are basically non-refundable. A little inhuman!!! It is also artificial scenery, the parking lot to the entrance, the linear distance is estimated to be 1 km, set up countless barricades drainage, I hold the child, got off the car to go how also have three kilometres, holding the child, did not go to the parking lot ferry battery car! And then there’s the crap about creating an express lane that I thought everyone could grab, which is a queuing app, but it’s not, it’s limited, it’s a queue-cutting app, it’s a blatant queue-cutting app. Anyway, I’m never coming back!!!

By checking the representative comments, we found that negative comments are directed to the following issues: (a) the parking fee is quite expensive, (b) the parking lot is far from the entrance to the theme park, (c) the parking lot environment is poor, and (d) there is no traffic control in some parts of the parking lot, such that the comprehensive management is poor.

The second top negative topic regards Service Attitude. Below, we show a representative review of the in-park services:

[Topic—Service Attitude] Come on, 500 words bad reviews are ready!!! I went to Disney first thing in the morning, and it took me a long time to get in. The security staff is rude, the staff inside is unresponsive to the tourists, and the restaurant service inside Disney Town is rude. What do you mean, don’t eat? You eat somewhere else. Inside the hamburger, French fries 119 servings, the amount is small, but also bad taste, there is no place. Go to what rubbish Disney bar, money does not say, the experience is very bad, the service staff attitude is very, very bad, it is not as good as Tianjin Happy Valley. Anyway, I don’t want you to bring your kids. If you really want to go, weekdays to experience how bad, don’t go to the holiday such as Chinese New Year, affect the mood.

The online reviews that fall into this topic cover a number of areas: (a) the inefficiency of purchasing tickets, (b) the ‘blank eye’ given by service staff to customers, (c) staff shouting at customers, (d) the low quality of service staff, and (e) the violent unpacking. The topic that ranks as the third is Recommending Feeling. Upon reading some representative negative comments, we drew conclusions that several themed areas in Disneyland are strongly recommended by tourists, but some amusement facilities (e.g., the pirate ship and food service) did not meet their expectations and are thereby not recommended.

**Figure 2:**
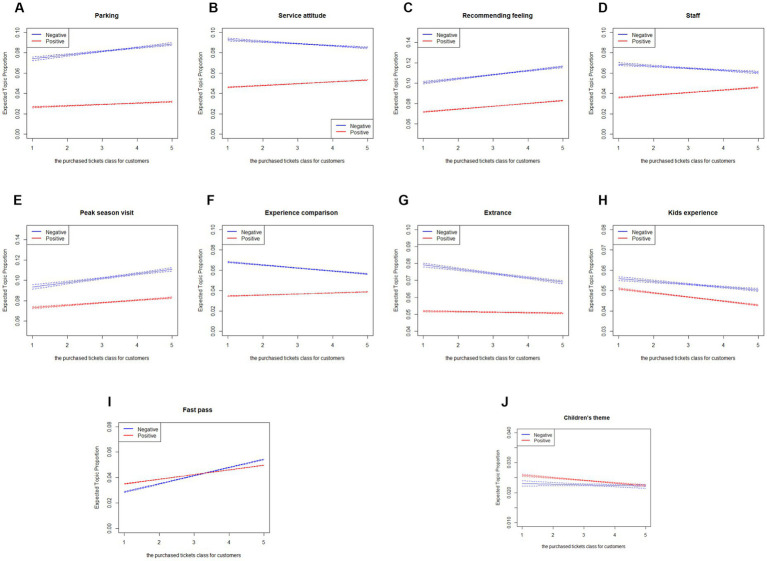
**(A–J)** Effect of different customer types (the purchased tickets) on topic proportions. (1) The 1, 2, 3, 4, and 5 of *x*-axis represent children ticket, student ticket, adult ticket, family ticket, and other ticket, respectively. (2) The *y*-axis topic proportion represents the percentage of topics among all reviews.

To further explore whether customer types had different aspects regarding dissatisfaction, this study categorized customers into five groups based on the tickets purchased. We coded the ticket types children ticket, student ticket, adult ticket, family ticket, and other ticket as 1, 2, 3, 4, and 5, respectively. These codes were then used to represent the five customer types. Next, the changes of the topic proportion under the five categories were plotted in Figure 2, where the *x*-axis and *y*-axis represent the purchased tickets for customers and the expected topic proportion.

From Figures 2A,C,E,I, we found that the proportion of the four topics in the negative comments increases with respect to the number of tickets purchased by customers. Taking the topic Fast Pass as an example, its proportion in the negative comments increased from 3 to 5.3%, and its proportion in the positive comments also increases as the types of purchased tickets. In addition, based on reading the representative comments, although officials have claimed that fast passes are free, several tourists report that they are still charged and relatively expensive and that “fast track” still has queues in many cases. As such, it can be concluded that customers who purchase children tickets are more concerned about when the rides can be accessed, while those who purchase family tickets place more emphasis on the fast pass service.

Figures 2B,D,F,G,H show that the proportions of five topics (Service Attitude, Staff, Experience Comparison, Entrance, and Kids Experience) decrease in accordance with the tickets type changes from 1 to 5. For example, for the topic ‘Entrance’, its proportion of negative comments decreases from 8% (children ticket) to 6.8% (other ticket). From the representative comments of the topic, a number of key insights are outlined: (a) the time taken to enter the theme park, (b) whether the fast pass is effective and (c) whether ID cards are required to enter the park. Such topics suggest that Shanghai Disney Resort failed to control the flow of people entering the park, and many of the admission processes could be managed. Furthermore, for the fast pass admission, tourists may feel psychologically unbalanced about spending money again on the entrance process (Pritchard, 1969), thereby leading to dissatisfaction.

Based on the analysis results of the negative topics, we identified two main reasons behind the negative experience. First, too many tourists decrease the number of facilities they can access per visit, which leads to negative feelings about the service, especially when queuing. Second, failure to meet the expectations of tourists on some services such as fast pass admission could largely affect their overall experience even if they have fun at the amusement facilities. In addition, customers who purchased different tickets had some differences in the identified aspects.

## 4. Conclusion

This research aimed to explore the aspects that lead to dissatisfaction in theme park visitors and whether different types of customers may care about different aspects in respect to their dissatisfaction. We utilized the STM approach to identify the negative topics from the online customers reviews on Shanghai Disney Resort. Several conclusions are drawn in this study. Firstly, this study answers the key questions about tourists’ dissatisfaction with the theme park, which extends the existing literature on theme park studies. We have revealed that the problems of “Parking,” “Service attitude,” “Recommending feeling,” “Experience comparison,” and “Entrance” could be the main reasons for tourists having a less enjoyable experience.

Secondly, this study provides a novel perspective to explore the relationship between different types of purchased tickets and their effect on the proportions of these topics. We found that when tourists were travelling in a group (e.g., with a family ticket), customers felt less enjoyment due to parking problems and fast pass issues. In contrast, when tourists were travelling alone or with a small group, the problems of service attitude amongst staff, experience comparisons, and admission processes were of greater concern. Our findings could help the managers of Disneyland focus on the areas identified as negative in this research, thereby permitting them to provide their customers with better services and experiences.

Despite the several valuable insights obtained by this research, there are limitations that could be explored in future research. Firstly, this study examined only one theme park (Bai et al., 2023); therefore, the conclusions may be limited to a certain context. Future research can be extended to multiple theme parks. In addition, only the textual comment data were analysed. In future research, we will utilize several types of data (such as visual content) to reveal and further explore more interesting and important phenomena and findings (Yang et al., 2021).

## Data availability statement

The data analyzed in this study is subject to the following licenses/restrictions: the data of this study is obtained through python-based web crawler. Requests to access these datasets should be directed to HH, he156958505@163.com.

## Author contributions

SB and HH: conceptualization. MY and HH: methodology. XB: formal analysis. HH: writing—original draft. HH and WF: data curation and software. HH and CH: writing—review and editing. MY and CH: supervision. SB: project administration and funding acquisition. All authors contributed to the article and approved the submitted version.

## Funding

We appreciate the financial support of the Reform and Develop High-Level Talent Projects in Local Universities Supported by the Central Government (Grant No. 2020GSP13) and the Natural Science Foundation of Heilongjiang Province (Grant No. JJ2021LH1530).

## Conflict of interest

The authors declare that the research was conducted in the absence of any commercial or financial relationships that could be construed as a potential conflict of interest.

## Publisher’s note

All claims expressed in this article are solely those of the authors and do not necessarily represent those of their affiliated organizations, or those of the publisher, the editors and the reviewers. Any product that may be evaluated in this article, or claim that may be made by its manufacturer, is not guaranteed or endorsed by the publisher.

## Footnotes

^1^Available at: www.meituan.com/zhoubianyou/93311902/.

^2^Available at: https://github.com/fxsjy/jieba.
